# The persistent power of stigma: A critical review of policy initiatives to break the menstrual silence and advance menstrual literacy

**DOI:** 10.1371/journal.pgph.0000070

**Published:** 2022-07-14

**Authors:** Mary M. Olson, Nay Alhelou, Purvaja S. Kavattur, Lillian Rountree, Inga T. Winkler

**Affiliations:** 1 Institute for the Study of Human Rights, Columbia University, New York, New York, United States of America; 2 Columbia University, New York, New York, United States of America; 3 Department of Legal Studies, Central European University, Vienna, Austria; University of Essex, UNITED KINGDOM

## Abstract

Menstruation is shrouded in stigma and shame—that is the common refrain in burgeoning initiatives on menstrual health and hygiene. Public policies alone cannot undo stigma and enact social change, but they do interact with social norms. They can reflect and adopt stigmatizing attitudes and, as a result, institutionalize, formalize, and legitimize stigma; or they can actively challenge and denounce it and mitigate existing discrimination. Against this background, we explored whether and how policies on menstrual health and hygiene address menstrual stigma and advance menstrual literacy based on an analysis of 34 policy documents and 85 in-depth interviews with policy-makers and advocates in four countries: India, Kenya, Senegal, and the United States. We found that policies *recognized* menstrual stigma and set out to break the silence surrounding menstruation and advance menstrual education, but they did not contribute to *dismantling* menstrual stigma. Policy-makers seemed constrained by the very stigma they sought to tackle, resulting in hesitancy and missed opportunities. Policies raised awareness of menstruation, often with great noise, but they simultaneously called for hiding and concealing any actual, visible signs of menstruation and its embodied messiness. Educational initiatives mostly promoted bodily management and control, rather than agency and autonomy. As a result, policies might have succeeded in breaking the silence around menstruation, but stigma cannot be broken as easily. We first need to recognize its (invisible) power and its impacts in all spheres of life in order to actively challenge, dismantle, and redefine it.

## Introduction

Scholarship, programs, advocacy, and policy development on menstruation are burgeoning. Documentation of these efforts commonly start by pointing out that menstruation is surrounded by stigma and shame. For instance, a recent stock-taking paper on the state of menstrual health laments that “menstruation continues to be shrouded in silence and stigma and remains a neglected issue” [1]. Menstrual stigma refers to the negative perception of menstruation and those who menstruate [2], characterizing the menstruating body as abnormal and abject [3]. Indeed, stigma lies at the root of understanding why and how menstruation matters. It has significant impacts on all spheres of menstruators’ lives including their health, education, economic opportunities, and participation in public and social life [2, 4, 5].

Recent policy developments seek to disrupt these patterns and perceptions. They have grown out of a greater awareness of menstrual needs that has moved menstruation onto the global health agenda [6–8]. While some authors have offered assessments of policy developments—in particular in the United States and India [9–13]—to date, there is no empirical, comprehensive, cross-country analysis. Our study seeks to contribute to filling this gap through an analysis of policy documents and extensive interviews with relevant stakeholders. As part of a broader project, this paper focuses on examining efforts to raise awareness and advance menstrual education.

Policies publicly—and officially—address menstruation with the explicit objective of raising awareness, thus bringing this “private” matter into the public. For example, the Kenyan Policy on Menstrual Hygiene Management (MHM) seeks “to ensure that myths, taboos and stigma around menstruation are addressed by providing women, girls, men and boys access to information on menstruation” [14]. Policies have the potential to destigmatize and normalize menstruation. Not only does a policy indicate a government’s willingness to address menstrual needs, but it also signals that menstruation is (or should become) part and parcel of public debate.

As more countries address menstruation as an issue of public policy, our project focused on reviewing policy initiatives related to menstruation in four countries: India, Kenya, Senegal and the United States. We explored the processes, triggers, drivers, and opportunities that have led to the elevation of menstrual hygiene and health at the policy level and the resulting framing and focus of policies. As part of a broader study, this paper explored to what extent policies address menstrual stigma and advance menstrual literacy. We critically examined whether they succeed in triggering the much-needed upheaval to disrupt the status quo of the pernicious, deeply entrenched control of menstruators’ bodies.

We found that policies set out to address menstrual stigma by “breaking the silence” surrounding menstruation and advancing menstrual education, but they do not reach far enough. Policy-makers seem constrained by the very stigma they seek to tackle, resulting in hesitancy and missed opportunities. Policies raise awareness of menstruation, often with great noise, but they simultaneously call for hiding and concealing any actual, visible signs of menstruation and its embodied messiness. As a result, they inadvertently perpetuate the very stigma they seek to erase. Educational initiatives promote bodily containment, management, and control, rather than agency and autonomy. Yet, to dismantle invisible menstrual stigma we need to first acknowledge its power and go beyond menstrual management to promote comprehensive menstrual literacy that recognizes menstruating individuals as decision-makers based on agency and autonomy.

## Menstrual stigma

To analyze the selected policies, we first need to understand menstrual stigma, its impacts and workings, and the role of public policy in addressing stigma.

### The impacts and workings of menstrual stigma

Menstrual stigma can significantly affect personal, social, and economic well-being. As with all forms of stigma, the impacts are felt not just in one domain, but across all spheres of life [15]. Stigma hinders discussions about menstruation. Due to a lack of reliable, accessible information, many people have misconceptions about why menstruation occurs [16, 17]. This results in a lack of understanding—of the menstrual cycle, of biological processes, and of meanings ascribed to menstruation, which would help make sense of sociocultural practices and contexts [1].

Menstrual stigma has lasting implications. It results in reinforcing misogynist stereotypes, with menstruators being perceived as “irrational,” “too emotional,” “hysterical”—and as a result, less capable [18, 19], which influences participation in public life and economic opportunities. In a psychological study, women who signaled their menstrual status by dropping a tampon in front of others were ranked less competent and less likeable than someone who dropped an object perceived as neutral, such as a hair pin [20]. Teasing and bullying because of menstruation are significant problems for girls’ educational experiences and outcomes [21]. As a result, menstrual stigma significantly restricts menstruators’ daily social and physical activities [16]. Menstrual stigma also has health consequences. Bias against menstruating bodies is persistent in medical textbooks, and this may carry forward as health professionals work with menstruators [22]. While conditions related to menstrual health are manifold, endometriosis provides a glaring example: The delays and gaps in diagnosis, care, and treatment often mean menstruators wait years for a diagnosis, and in the meantime their pain is dismissed or misdiagnosed [23, 24].

These impacts range across all facets of menstruators’ lives, yet they all relate back to power relationships in a society characterized by sexism and misogyny. Menstrual stigma carries an invisible power to define what is normal and acceptable—and what is not. The hairpin is; the tampon is not. A broken bone is; endometriosis is not. Education on nutrition is; menstrual literacy is not. Menstrual stigma is so pervasive and deeply embedded that it remains largely invisible. In fact, a recent paper creating a taxonomy of 93 stigmas does not even cover menstruation [25]. As a result, menstrual stigma is (invisibly) embedded in the social dynamics which (re)produce unequal power structures that impact the realization of human rights. Stigma serves to “[fortify] existing social hierarchies” and as such provides a rationale for discrimination so that discrimination is seen as natural, necessary, and desirable [26].

Sociocultural norms reinforce menstrual stigma through imperatives of concealment and hygiene, which cultivate the idea that menstruation is shameful and should therefore be hidden, controlled, and managed [19]. In societies characterized by sexism, misogyny, and somatophobia, women are faced with (impossible) norms and standards. As Elizabeth Grosz explains, “misogynist thought has commonly found a convenient self-justification for women’s secondary social positions by containing them within bodies that are represented, even constructed, as frail, imperfect, unruly, and unreliable” [27]. The bloody, leaky, messy process of menstruation, in particular, is considered “dirty, disgusting, defiling” [19]. Bodily fluids flow, leak, and seep—the opposite of being clean and proper [27]. Menstruating women are considered abject and “out of control” [19]. Iris Marion Young explains that “the normal body, the default body, the body that every body is assumed to be, is a body not bleeding from the vagina. Thus to be normal and to be taken as normal, the menstruating woman must not speak about her bleeding and must conceal evidence of it. The message that the menstruating woman is normal makes her deviant, a deviance that each month puts her on the other side of a fear of disorder, or the subversion of what is right and proper” [19].

Because this proper body is coded as male and non-menstruating, menstruators employ what Sharra Vostral terms “technologies of passing,” menstrual products that serve to hide and conceal and allow their users to present and represent themselves as non-menstruating [28]. The management of menstruation serves to reinstate control over a body that is seen as out-of-control. Menstrual products are often advertised to conceal odors and ensure that menstruators stay “fresh” [2]. This insistence on “fixing” any visible, smellable, or even audible [29] sign of menstruation perpetuates the notion that menstruation is unclean, abnormal, and abject. The resulting concealment imperative places the burden of managing one’s stigmatized body on the individual. In addition, menstruators feel pressured to avoid the subject of menstruation. Discussions about menstruation are often considered private, typically restricted to conversations with female family members; if menstruation is addressed publicly, it is only through euphemisms [30]. These imperatives of concealment are regulatory practices working through shame and stigma that serve to discipline and contain “the monstrous feminine” [31].

The containment and concealment make addressing stigma all the more difficult, as menstruators feel pressured to hide any cue that they may be a part of the stigmatized group. As a result, menstruators often internalize stigma, which can have significant impacts on their self-esteem and cause increased self-monitoring, hypervigilance, and possibly limited participation in society [32]. The concealment and hygiene imperatives developed because of stigma; and in response to stigma, they provide coping mechanisms for menstruators and render menstruation invisible. As a result, stigma becomes deeper ingrained in society.

### Menstrual stigma as a matter of public policy

Menstrual stigma is socioculturally constructed, which implies that it can also be deconstructed by changing sociocultural norms. These norms are influenced by multiple actors and forces, and public policies interact with these norms and actors. To be sure, we cannot simply legislate or enact social change through policies—norm evolution is a complicated transformation. Examples from other countries show that sociocultural practices do not change simply because a law criminalizes them [33]. Dismantling menstrual stigma is a multilayered, long-term process that involves all actors in society. Yet, policies play a crucial role in either reinforcing or mitigating stigma. Government action that challenges menstrual stigma—such as information campaigns and education—can be useful in promoting norm evolution [34]. Policies define expected behavior; they can encourage and incentivize. They provide the forum to interrogate social norms, and the language they use sends powerful signals. Policies can reflect and adopt stigmatizing attitudes and, as a result, institutionalize, formalize and legitimize stigma; or they can actively challenge and denounce it [35, 36], mitigate and interrupt its impacts, and protect people from the resulting discrimination [15].

## Research design and methodology

To comprehensively review menstrual hygiene and health policies in four countries, we analyzed existing policy documents and conducted in-depth qualitative interviews with policy-makers and policy advocates on menstrual hygiene and health policy. We followed the COREQ and SRQR guidelines to report on data collection and analysis [37, 38].

We selected India, Kenya, Senegal, and the United States based on their leading roles in the field of menstrual health and hygiene policy and on their ability to provide geographic diversity. South Asia and East Africa, and subsequently North America and West Africa have seen early and extensive policy developments in the last decade [3, 9]. Within these regions, our selected countries are early adopters: Kenya removed value added tax on menstrual products as early as 2004, with other policies following from 2011 onwards. India adopted the first relevant policies in 2009, while integrating menstrual hygiene into the Total Sanitation Campaign in 2013 prompted the issue to gain significant attention. Senegal and the United States (including New York City) adopted the first policies in 2016. The earliest initiatives were concerned with the taxation and provision of menstrual products and relevant infrastructure and were followed by additional policy developments (for more details see the S1 Table) [39]. We conducted our review at the national level and a selected region (state, county, and/or municipality) to gain insights into implementation strategies. Selected regions were Maharashtra in India, Kwale County in Kenya, the Louga and Diourbel regions in Senegal, and New York State and New York City in the United States. As with the countries themselves, we selected these regions based on their being early adopters.

We reviewed 34 policy documents from India (n = 10), Kenya (n = 9), Senegal (n = 4), and the United States (n = 11) (complete list available in S1 Table). We relied on a number of sources to identify the documents, including the project’s Advisory Group, relevant stakeholders, and the collection of policies in the Menstrual Health Hub [40]. Most documents were adopted at the national level, while some legislation and policies in the United States were developed at the state and municipal level. We included documents that (a) covered a topic related to menstruation, (b) were adopted between 2000 and 2020, and (c) comprised a policy enacted by a governmental entity, including legislation and government-approved guidelines or mandates.

From April to October 2020, we (PSK and NA) conducted interviews with 85 participants in India (n = 19), Kenya (n = 19), Senegal (n = 23), and the United States (n = 24). One additional interview from Senegal was not included in the analysis because the participant did not provide their informed consent form despite follow-up. We used the video-conferencing software Zoom to conduct and record the interviews in private and confidential remote settings. In most cases, we conducted them individually, except where participants preferred a group setting. All participants signed an informed consent form and were informed during interviews about the interviewer’s background and research objectives. Except for arranging interviews and brief email exchanges, the interviewers (PSK and NA) did not have any previous contact with the participants, whereas ITW knew some of the participants from previous work on menstrual health. Interviewers followed a loose script of questions on major research themes. They focused on the processes, stakeholders, institutions, and priorities for policy development and implementation. Questions revolved around: Which aspects of menstruation do policies address? What are their objectives? How do they seek to address menstrual stigma? What strategies were put in place for menstrual education? How do they advance menstrual literacy among different groups? (Interview guide available in S1 and S2 Texts).

Interviews in India, Kenya, and the United States were in English. In Senegal, most interviews were in French, and the remainders were conducted with the assistance of an interpreter. We translated French quotes into English. The interviews lasted one hour on average but ranged from 30 minutes to 2 hours. All interviews were transcribed to prepare them for data analysis and assigned a code to ensure anonymity.

We selected participants based on their active involvement in policy-making or policy advocacy. They included government officials at different levels, members of civil society organizations, social entrepreneurs, staff in NGOs, foundations and UN organizations, academics, and other experts on menstrual health and hygiene. Due to their positionality, government interviewees might have had an overly positive assessment of policies they were involved in. We took several measures to mitigate the risk of biased responses: We ensured anonymity in order to enable all participants to speak freely. We interviewed several former government officials who have gained some distance from the initiatives they were involved in. Finally, we deliberately identified interview partners engaged in the same processes from different stakeholder groups to enable us to gain different perspectives.

Our selection process included a number of strategies: (a) a careful review of relevant literature and documents to identify referenced stakeholders; (b) initial contacts in the menstrual hygiene and health space as well as in adjacent policy fields such as human rights, labor, gender, education, and sexual and reproductive health and rights; (c) introductions by members of the project’s Advisory Group; and (d) snowballing. We deliberately interviewed participants working on diverse issues ranging from menstrual hygiene initiatives to endometriosis advocacy; and we also sought to interview advocates with disabilities, trans advocates, and people working in informal settlements, among others, to ensure diverse voices. In many cases, we were referred to the same people and organizations repeatedly, indicating that we had identified the key stakeholders.

Based on the model set out by Virginia Braun and Victoria Clarke [41], we conducted a qualitative thematic analysis of the policy documents and interview transcripts. Following a deductive approach, we developed a codebook based on (a) a close reading of the policies, (b) the initial completed interviews, and (c) our own knowledge of the field. The codebook included codes related to policy issues, actors, policy cycle stages, and target populations. It covered 36 distinct issues, including taxation, facilities and infrastructure, disposal, education, awareness raising, addressing stigma, and various aspects of menstrual health. In this paper, we focus on policies related to stigma, awareness raising, and education. A team of five coders (PSK, NA, LR, MMO, and a research assistant) conducted the analysis using NVivo 12. To ensure the internal validity of coding and thematic synthesis, the coders double-coded a sample of transcripts prior to the analysis and met to discuss and reconcile any discrepancies.

Our study was approved in its entirety by the Institutional Review Board of Columbia University (Protocol Number: AAAS8659). We received additional national ethical clearance for our research in Kenya and Senegal. No ethical clearance was requested in India, as only biomedical and clinical studies require it. In Kenya, Amref’s Health Africa’s Ethics and Scientific Review Committee cleared our research (Protocol Number: P775 2020), and the National Commission for Science, Technology and Innovation provided a research permit (License Number: NACOSTI/P/20/5059). In Senegal, the Comité National d’Ethique pour la Recherche en Santé approved our protocol (Protocol Number: SEN20/40). Additional information regarding the ethical, cultural, and scientific considerations specific to inclusivity in global research is included in the S1 Checklist.

## Findings: Awareness raising and education

Menstrual health and hygiene policies covered a range of issues, from providing menstrual products and building appropriate facilities to ending taxation of menstrual products. The predominant policy focus was on tangible and material solutions, but there was also an acknowledgments that many of the challenges related to menstruation are intangible and rooted in stigma [39]. Of the policies we reviewed, 7 out of 34—all from India and Kenya—explicitly addressed menstrual stigma. No policies from Senegal or the United States explicitly discussed stigma, but policy-makers and advocates acknowledged it as a challenge. In addition, about one-third of the policies addressed menstrual education, which creates awareness. Information is power and literacy the best antidote to stigma, so these policies also have the potential to address menstrual stigma.

Kenya’s MHM Policy and India’s MHM National Guidelines were the most thorough in outlining objectives to address stigma. They identified two deeply intertwined problems: silence and a lack of information. “Silence about menstruation is a universal problem contributing to widespread stigma” [14], Kenya’s MHM Policy states; and India’s MHM National Guidelines identify that “there is [a] lack of information on the process of menstruation…. The taboos surrounding this issue in the society prevent girls and women from articulating their needs” [42]. These two aspects create a vicious cycle: Because there is silence, there is a lack of information; and because misinformation presents menstruation as shameful, there is silence. The policies noted that combatting menstrual stigma is necessary “to enable … women and girls to access information, make informed choices and participate fully in all walks of life every day of the month” [14]. As such, raising awareness about menstruation was linked to ensuring participation in public life.

In the following, we will present policy efforts to break the silence around menstruation, to engage men and boys, and to advance menstrual education. In each subsection, we will first introduce various policy initiatives and then share the critiques that some interviewees offered, which will provide the basis for our discussion and recommendations for future policy-making in the next section.

### Breaking the silence

Initiatives to break the silence around menstruation—among people who menstruate, the general public, and policy-makers—were common throughout the four countries and characterized much of the rhetoric. For instance, “Breaking the silence on menstruation” was one of the three key prongs of Kenya’s strategy [14].

Policies aimed to create spaces to freely discuss menstruation. “The way we try and break this whole culture of shame and silence is we sit with women and we talk to them about [menstruation], we actually engage with them and ask them to open up the subject and get them to speak,” an interviewee in India noted [IND06]. Interviewees in Senegal explained how girls benefit from a space in which they can talk freely.

Because they were suffering and they didn’t know who to talk to, because this was kind of taboo, and they were ashamed to talk about that with a teacher or their mother. But with their laboratories [workshops], they freely talked about what they felt [SEN08].

One of the key activities was sensitization trainings targeting a range of populations, including community members, teachers, students, ministerial staff, and policy-makers. One interviewee explained, “Our first step consists of sensitizing the political authorities and decision-makers and then the technicians.… We needed to talk at the level of departmental ministries, so the Ministry of Water and Sanitation, the Minister of Health, the Minister of National Education, the Ministry of Environment” [SEN16]. Sensitizing governmental officials was considered important because “for menstrual hygiene management to become something successful in Senegal, we need political will” [SEN14].

Exposing policy-makers first-hand to menstrual experiences has been key in raising awareness. Field visits and direct engagement provided a platform for menstruators to share their experiences and concerns with policy-makers—for instance, during the Great WASH Yatra in India. One interviewee claimed,

The non-negotiable components … are definitely direct voices.… You have to bring menstruators to talk and share their experiences with politicians and bureaucrats.… And we did that successfully. And we didn’t just do that with one or two. We did it with 12,000 women and girls across five states [in India].… And the bureaucrats listened [IND02].

Community celebrations also enabled discussions with constituents. For example, the “MHM mela” was a festival and gathering across several villages in Maharashtra, India, that offered movie screenings, pad demonstrations, and other activities. One interviewee recalled that the “mela itself was an eye-opener for a lot of block level officials” [IND19]. Individual efforts, such as letters to officials from menstruating constituents, were similarly successful in raising awareness. A legislator in the United States remembered: “One day, a few years ago, a junior high school student wrote me a letter about how people who live in New York City homeless shelters were not able to get these products” [USA15]. Hearing from this student provided the initial trigger for the change in regulations of the Federal Emergency Management Agency to cover menstrual products [USA15]. In many countries, Menstrual Hygiene Day, on May 28^th^, creates a space to raise awareness on social and mainstream media and to reach the general public. When it began in 2014, “we had around 35 grassroot organizations and small enterprises that came together,” recalled one Kenyan interviewee [KEN02]. Over the years, more and larger organizations have become involved, and Menstrual Hygiene Day has turned into a rallying point for attracting attention to menstrual hygiene [KEN03].

This model of awareness raising is based on the assumption that talking about a stigmatized issue will end the stigma. However, several interviewees questioned the reach and impact of initiatives to break the silence. One interviewee in Kenya challenged the idea that having stakeholders talk about menstruation was enough to enact change: “At lunch people talk to each other about it, but … then everybody carries on with the day” [KEN12]. Overall, it remained unclear exactly how breaking the silence translates into concrete action or attitudinal change. For example, the Indian MHM National Guidelines included the welcome acknowledgments that

Menstrual hygiene management is a social issue that cannot be addressed by working in schools alone. In order to ensure that adolescent girls and women have the necessary support and facilities, it is important that the wider society, communities and families must *challenge the status quo and break the silence around menstruation* [emphasis added] [42].

The policy continued: It is “the responsibility of those with influence—including government officials and teachers, to find appropriate ways to talk about the issue and take necessary actions” [42]. Yet, it did not specify these “appropriate ways” and “necessary actions.” While one could argue that policies should leave space for innovation without dictating micro-level solutions, the Guidelines could and should include suggestions. While policies were very specific in relation to providing menstrual products, solutions to address the overarching objective of reducing menstrual stigma remained vague and limited. The policies rarely identified what breaking the silence entailed, nor did they spell out steps to be taken *after* the silence has been broken.

### Engaging men and boys

Policies and interviewees across countries recognized the need to raise awareness among men and boys and to engage them in advocacy and education. Non-menstruators play key roles in shaping menstrual experiences as supportive or nonsupportive family members, friends, and classmates (including as potential bullies) [21]. They often serve as decision-makers on issues ranging from household purchasing to policy development [43]. In response, India’s MHM Guidelines stressed, for example, that men and boys must be targeted in information campaigns “because they need to positively support their sisters, daughters, wives, aunts and mothers” [42]. Almost half of the interviewees (39 out of 85) mentioned the role of men and boys. One interviewee stressed that it has become “very, very clear that everybody has a role in the whole process” [KEN16]. In Senegal, men were targeted through community sensitization trainings [SEN06]. Similar efforts were underway in Kenya. In Kwale,

we have outreach campaigns whereby we go with our clinic and we have open discussions with different groups. We talk to men, the fathers, we talk to the mothers, women, we talk to the children, both boys and girls and there’s openness. And more often I have had fathers coming out to openly say that I wish I had someone telling me about these issues earlier [KEN05].

Similarly, in India,

where we feel that the community is willing and is supportive, that’s where we bring in men and boys, into the conversation because we feel that they have to be those champions, they have to support and they have to provide that enabling environment for women and girls when it comes to prioritizing their health and well-being [IND13].

One interviewee explicitly argued that “there is need to involve boys … so that there is no stigma. And also, to ensure acceptance and avoid rejection or any frustrations which would psychologically impact those young girls” [SEN04]. Interviewees also noted gender-based purchasing power disparities at the household level and stressed the need to promote monetary allocations toward menstrual products [IND04]. Finally, interviewees emphasized the continuing outsized role of men as policy-makers. In the United States, for example, there was a noticeable knowledge gap between menstruating and non-menstruating legislators’ responses to policy efforts. One interviewee in the United States shared: “So that was this big aha moment where our team realized … that there were all of these areas because all of these bills had been written by men that menstrual hygiene products just weren’t even considered” [USA19]. In response, advocates have engaged prominent male figures as public allies [44].

The emphasis on engaging men and boys implicitly acknowledges patriarchal power relations at all levels of society: the power of male household members to determine purchasing priorities, the power of school bullies, and the power of policy-makers. Yet, very few interviewees stated explicitly that stigma is about power relations. An exception was an interviewee from India who briefly acknowledged the power dynamics in the intersection of menstrual stigma, gender, and caste [IND03]. Another recognized the role of “patriarchs, or the head of families, or the men in the families who have power, and there are power dynamics in family. We cannot assume that they are not there. These power dynamics play a role” [IND04]. Looking beyond household dynamics, another interviewee in India explained:

I believe if you want to [fight] strategically on the certain barriers of MHM, which is linked with the religion and theology, we need to fight it strategically, bringing the judiciary in the system, bringing the doctors in the system, bringing the social worker in the system [IND17].

Yet, these acknowledgments were the exception, and no interviewee explored the workings of the power of stigma in more depth.

### Educating on menstrual management

In addition to raising awareness, policy initiatives in all four countries promoted menstrual education. Providing information and knowledge was seen as the primary way to tackle menstrual stigma, which is driven by misogyny, myths, and misinformation. Of the 34 policies we reviewed, over one-third sought to advance menstrual education. Government officials recognized the importance of menstrual literacy. While the main focus was on girls in schools, some policies targeted a broader audience. Overall, such trainings aimed to demonstrate that menstruation is “not a shame, it’s not a disease, it’s not something to hide” [SEN15].

Menstrual education initiatives largely focused on menstrual hygiene and the use of menstrual products—the practical elements of managing menstruation. One interviewee in Senegal explained that they started

awareness building in the regions with a product that we already had, which is reusable sanitary pads. And we toured the regions and villages showing these sanitary pads, and we realized that there were recurrent questions from women about how to use it or how to manage the hygiene thereof [SEN07].

Such remarks demonstrated how intertwined menstrual education is with the promotion of menstrual products. In Senegal, many interviewees argued that sensitization trainings were needed to teach women and girls how to use menstrual pads and other materials. One government official claimed that women and girls

mismanage [their] period because quite simply today, when she goes to school, she is embarrassed, she hides, she takes her pads, she throws them somewhere because she is not well informed. She’s going to pollute the atmosphere, she’ll clog the toilets. She doesn’t know what to do. She is lost [SEN13].

One of the main motivations driving sensitization trainings was enabling girls to avoid using “inappropriate material to manage their menstruation” [SEN02]. Similarly, an interviewee in India pointed out that “critical behavior change … is around products and disposal” [IND13]. A Kenyan interviewee argued that information on product use and disposal is crucial “so that then people are able to understand what menstrual hygiene management is all about, what it entails in terms of the needs, what it entails in terms of disposal, what it entails in terms of the products” [KEN16]. Another interviewee in Kenya referred to menstrual hygiene products as “special waste” [KEN13], which reinforces the idea of menstruation as dirty and polluting. The overall message is that menstruators pose a risk and need to learn to manage their menstruation *properly* by using *appropriate* materials.

Policy documents and interviewees frequently pointed to the embarrassment and shame menstruators feel when leaking, smelling, or staining. For example, the Swachh Bharat Mission states: “Women suffer in the absence of knowledge about safe practices on MHM” [45], which encapsulated the typical educational information: how to manage menstruation. In response, the Indian MHM Guidelines stated that “Hygienic menstrual absorbents help adolescent girls to manage menstruation effectively, safely and comfortably. Freedom from the fear of leakage or unpleasant odour increases a girl’s ability to be at school during menstruation” [42]. The Kenya Sanitary Towels Programme similarly justified its mission by stating that “The inaccessibility of menstrual products [results] in embarrassment, anxiety and shame when girls and women stained their clothes” [46]. In Senegal, one interviewee explained that “some ignorant teachers humiliate [girls] when they happened to have their menstruation in the classroom. They were told that they had to be more cautious. And they were being mocked by their classmates” [SEN05]. As “leakage” or “unpleasant odor” are stigmatized signs of menstruation [2], the rationale is that having access to products and facilities can help avoid such scenarios and curb embarrassment. The suggested solution, then, was providing menstrual products and constructing toilets because “girls needed a whole set of facilities to manage their menstruation” [SEN05].

The focus on menstrual products as a solution was reflected in and reinforced through budgetary and spending priorities. One informant in Kenya lamented, “apart from the free sanitary pads program, I’m yet to see the government at least showing commitment by allocating some funds, even around awareness raising to break the silence. I’m yet to just see that” [KEN09]. In Indian policy initiatives, budgetary allocations for awareness raising and education were limited, and such education efforts “rarely mention tackling the socio-cultural taboos that play a critical role in how girls manage their periods” [10]. An Indian interviewee explained that “the awareness component started taking a slight backseat … when [district administrators or their wives] started getting more invested in this and they took it more towards pad distribution” [IND01].

Even where funding was allocated to awareness raising and education, budgets remained underutilized. The Swachh Bharat Mission in India came with significant resource allocations, and thanks to an amendment these funds could be used for menstrual hygiene [47]. A significant portion of the funds—8% of the total—was reserved for “social and behaviour change communication costs” [48, 49]. One Indian interviewee explained: “The Ministry had huge resources for what they call IEC, Information, Education, Communication, Training, Budgets. And, by 2016, that was two years into Swachh Bharat Mission, they had spent only 1% of that.… They should use that money” [IND08]. A former government official added: “It is still very uncommon to find blanket large-scale media on menstrual hygiene. And that again is one of the failures of our advocacy” [IND18]. An impact evaluation of the Swachh Bharat Mission estimated that only 2% of the total expenditure was used for informational and educational activities [48, 49]—a missed opportunity to use resources that had already been allocated to develop more comprehensive educational initiatives.

Several interviewees recognized that material solutions focused on the hygienic management of menstruation alone were not sufficient. By framing menstruation as in need of hygiene interventions, policies may have reinforced stigma and left out “a lot of the aspects of stigma and education, and identity” [USA17]. One government official in Senegal explained, “Even if … access to water and sanitation is addressed but the taboos are still here, if stigma is still here, women will never use these sanitation facilities, they will not speak about it” [SEN20]. In Kenya, one interviewee put it plainly: “Unfortunately, the hygiene aspect was in a way reinforcing the myths and the stigma itself” by labeling menstruation as dirty and in need of proper hygiene [KEN02]. Another interviewee in India lamented:

It’s an entry point, so why spend more time just issuing more guidelines on how to do this in a safe, sanitized manner? I mean, do we think women don’t know that now?… I think it’s a very safe way to raise donor money, but I would say it’s a bit shameful now that we’re still talking about these kinds of things, rather than looking at the complexities of menstruators’ lived lives [IND02].

### Expanding menstrual education?

Some programs and policies were more comprehensive and touched on aspects of menstrual health. In India, the Rajiv Gandhi Scheme for the Empowerment of Girls (SABLA Module) addressed the menstrual cycle and issues such as excessive bleeding, painful periods, and nutrition. It also described disorders such as amenorrhea and oligomenorrhea [50]. Such education can enable menstruators and those around them to reduce discomfort and to recognize when they need support. However, as one interviewee noted, “the scheme was small. It wasn’t implemented in all states and it wasn’t implemented in all districts” [IND01]. Moreover, studies reviewing SABLA have shown that these tools had mixed results both in girls’ understanding of menstruation and their comfort discussing it with friends, peers, and healthcare workers [51, 52]. Despite its goals, SABLA has not resulted in widespread, comprehensive menstrual education.

In the United States, puberty and sexuality education curricula are determined by each state. One interviewee lamented, “Sex ed isn’t required and when it is, it doesn’t include comprehensive education on periods” [USA23]. Some US states have started to address these gaps. New York State adopted a law regarding information on the signs and treatment of menstrual disorders, including endometriosis [53]. The legislator who worked on this bill lives with endometriosis herself and explained that she wanted to ensure that women do not have to wait years for a proper diagnosis due to the cycle of misinformation and stigma [USA04]. However, the act only stipulated that information be provided *upon request* [53], so dissemination was contingent on school actors’ knowledge of the law and their understanding that such education is necessary. Including it in the standard curriculum rather than making it optional would better guarantee that individuals receive the information they need.

In Kenya, at least some trainings were “not just [about] the product use, product disposal, product accessibility but also life skills because we realized that girls and also women going through menstruation face a lot of issues as far as their self-confidence comes in” [KEN18]. In Kwale County, interviewees identified their approach as “MHM plus”—indicating that MHM is the basis, with health information added on. They explained that they go with a team composed of the “officials from the health department. So that if there are any health-related issues that come up, issues related to reproductive health, they are there to respond and advise the community on these issues” [KEN05]. Their discussions encompassed reproductive health, ectopic pregnancies, pain, and teenage pregnancies, among other issues [KEN05]. Such instances show that menstrual hygiene programming can serve as an entry point to larger discussions of menstrual, sexual, and reproductive health when undertaken in collaboration with knowledgeable professionals who are prepared to address the questions that arise. Trainings and educational programs opened up spaces for discussions about menstrual health. Similarly, an interviewee in Senegal explained:

When you talk about MHM, they take advantage of that, they talk about their suffering relating to early menopause, endometriosis or other diseases. But we did not have general information to impart to girls and women. But as this is a medical, complex area, we refer them to medical assistance [SEN08].

People engaging in sensitization trainings clearly demanded menstrual health education, but healthcare providers were often not equipped to address these needs either [USA16; IND05; KEN08; SEN08]. One interviewee in India explained:

They don’t have any health-seeking behavior because of two reasons. One, because they don’t know. The question is, “I don’t know whether it’s bad enough that I need to go to the doctor,” because they don’t have the knowledge. The second thing is, they’ve gone to the doctor, and the doctor [has] gaslit them. “It’s all in your head” [IND04].

A lack of implementation, information that is only available upon request, and initiatives treated as add-ons all indicate missed opportunities to re-envisage menstrual education. In response, across all four countries, interviewees noted that better menstrual health education—encompassing an understanding of the menstrual cycle, various menstrual health conditions, and the way menstrual health intersects with gender identity—is needed. One interviewee explained:

You should know how your body works. That’s just basic.… It should be sort of a robust curriculum. But I think part and parcel, encompassed in that should be when things go wrong. Endometriosis, adenomyosis, fibroids, pelvic floor dysfunction. These things should all be addressed [USA16].

Interviewees noted the importance of educating adolescents about menstrual health conditions to curb diagnostic and treatment delays, as better health and body literacy allows menstruators to self-advocate during medical consultations [USA05; USA24]. This points to the need to strengthen menstrual health curricula and improve training for healthcare providers.

Overall, there was a tension between attempts to break the silence around menstruation and messages to cover it up. Menstrual education initiatives were largely limited to management and hygiene and rarely addressed menstrual health comprehensively. As a result, policies may have loudly raised awareness, but they shied away from encouraging (or even permitting) any visible signs of menstruation, as such (inadvertently) perpetuating the very stigma they set out to address.

## Discussion and recommendations

Policies addressing menstrual needs have advanced significantly over the last decade and have acknowledged that menstrual stigma presents a barrier to meeting menstrual needs. Policies sought to break the silence by engaging menstruators and non-menstruators and providing menstrual education. However, as several interviewees indicated, policies need to go further to undo menstrual stigma. These recommendations can be distilled into three categories: (a) going beyond menstrual management, (b) re-envisaging menstrual health education, and (c) recognizing menstrual stigma as patriarchal power.

### Going beyond menstrual management

The overarching focus of menstrual education in the policy initiatives we reviewed was on menstrual hygiene and management, which severely limited their potential to tackle menstrual stigma. To be sure, most people who menstruate prefer to use materials to catch the flow and will benefit from the provision of menstrual products. Most menstruators also prefer to care for their bodies in private—and we do not mean to dismiss progress made in enabling them to do so. However, our critique of current policy initiatives is twofold: The material solutions seem to drown out any other initiatives, and they are presented as a solution to address menstrual stigma. Rather than interrogating and challenging societal norms that ascribe embarrassment and shame to menstruation, menstruators are provided with menstrual products to cover up their shame. The lack of access to products does not *cause* embarrassment and shame (as the Kenyan Sanitary Towels Programme assumed) [46]; rather, social norms that present menstruation as dirty and shameful result in embarrassment. Messaging focused on hygiene and management calls for “appear[ing] in public without evidence of menstruation” and as a result “reinforce[s] ideas about … bodily and emotional self-control” [54]. Menstrual products allow menstruators to pass as non-menstruators both to others and to themselves, temporarily covering up the stigmatized condition that may negatively affect social interactions if revealed [28]. Passing serves as a coping mechanism, but it does not challenge the fundamental assumption that menstruation is shameful and the underlying misogynist attitudes [3].

The emphasis on concealment creates expectations and puts the responsibility on people who menstruate to manage, to keep clean, to exercise proper hygiene—to be “cautious,” as one interviewee put it [SEN05]. If a visible pad is viewed as problematic, then a stain or leak that could have been avoided is an even greater source of embarrassment and shame. As long as menstruation is seen as a “hygienic crisis” [55], it is not only presented as “a problem that *can* be fixed by the use of sanitary products” [56, emphasis added] but one that *must* be fixed. Ostensibly, the objective of breaking the silence is to make menstruation visible, but because awareness raising remains superficial, the predominant message remains management and concealment. As a result, stigma persists; it is covered up in layers of fluffy cellulose.

Menstrual products may certainly help to make menstruation visible. Openly carrying a tampon to the bathroom, washing a menstrual cup in a public sink, or drying a reusable pad on the clothesline in the sun can help normalize menstruation. Placing menstrual products outside of bathrooms can also serve to start a conversation around menstruation, in particular when combined with informational materials [57]. Menstrual artists such as Jay Critchley [58], Johanna Falzone, Phoebe Cing Ying Ma, and Ingrid Goldbloom use the materiality of menstrual products to make “the invisible visible” [59]. Maybe most powerfully, Judy Chicago’s photolithograph “Red Flag” depicts the removal of a blood-soaked tampon, making public an act that is usually carried out in private [60]. As such, the materiality, tangibility, and visibility of menstrual products give them the potential to serve as an entry point for tackling menstrual stigma. However, the focus of the policy initiatives examined here is quite the opposite: The messaging is that menstrual products serve to keep menstruation hidden.

We cannot expect policies alone to undo stigma and bring about societal change, but at a minimum they should not reinforce stigma, and they should contribute to normalizing menstruation—yet, they fall short of doing so. Even the use of the common term “menstrual hygiene management,” or its ubiquitous acronym MHM, presents menstruation as something technical and sanitized, devoid of its embodied messiness and its sociocultural meanings. To tackle stigma, policies must shift from presenting menstruation as something to be managed and controlled to developing a broad understanding of diverse menstrual needs. Policies need to identify and accommodate the needs of menstruators as menstruators at all levels of society. Campaigns that go beyond menstrual hygiene will require more complex and nuanced messaging, and lessons learnt can be gathered from public education campaigns in other areas such as HIV/AIDS and sexual orientation that have contributed to undoing stigma [36].

Dismantling stigma requires a reckoning with the public-private divide and gendered expectations of “respectability” and being “clean and proper.” Shilpa Phadke et al. show how these norms permeate public space, including a lack of accommodations for menstruation, and issue a powerful call for reclaiming public space [61]. Hygiene norms relate to the social order; they serve to “shore up the subjugated public roles of women” [36], which is evident in the imperative to conceal menstruation as outline above. “Women are expected to conform to the larger patriarchal order by demonstrating respectability,” [61] and as a result they perform appropriate femininity when accessing public spaces [61]. The use of menstrual products as technologies of passing then serves to stabilize systems of privilege and subjugation by ensuring that menstruation remains invisible [28].

We recognize that policies should continue to accommodate the immediate needs resulting from gendered expectations, such as enabling menstruators to take care of menstrual needs in private—we cannot put the burden on menstruators alone to challenge deeply entrenched societal norms by giving up privacy. At the same time, policy initiatives can and should contribute to shifting social norms; they should make us interrogate conceptions of respectability. Rather than providing the means to cover up “smelling, leaking, and staining,” they should help us realize a society where a spot of blood does not cause embarrassment but is just seen as something that happens to all of us who menstruate. While such attitudinal changes in the perception of menstruation require a long-term commitment, in the short term, policies must stop reinforcing the idea that menstruation is shameful.

### Re-envisaging menstrual health education

For policy initiatives to actually tackle menstrual stigma, they need to develop a deeper and more comprehensive understanding of menstrual health [62]. In our analysis, we identified a number of initiatives that attempt to advance menstrual literacy. However, the very efforts to dismantle stigma remain constrained by it, resulting in missed opportunities.

Menstrual education should be strengthened and expanded to cover the different aspects of menstrual health and menstrual experiences. Menstrual education can be effective in fighting stigma, but only if it goes beyond teaching the use of menstrual products. The aim must not be to enable menstruators to pass as non-menstruators, but to develop menstrual literacy as the basis for making informed choices. This requires “access [to] accurate, timely, age-appropriate information about the menstrual cycle, menstruation, and changes experienced throughout the life-course, as well as related self-care and hygiene practices” [62]. The menstrual cycle is considered a vital sign (alongside body temperature, blood pressure, pulse, and breathing rate), and menstrual awareness enables individuals to monitor other aspects of physical and mental health [63]. Menstrual education should cover pain by discussing strategies to ease discomfort while clarifying that not all menstrual pain is “normal.” It should bring awareness to menstrual disorders and health conditions. Such education should normalize menstruation without overmedicalizing it, acknowledging disorders and pain without portraying menstruation itself as a problem or disease.

Further, discussions of menstruation should integrate information about physical changes with discussions of the emotional and social aspects of menstruation [64]. Shobitha Rajagopal and Kanchan Mathur found that, in Jaipur, India, menstrual education programs focused too heavily on medical aspects without addressing the stigma menstruators experience [65]. Menstrual health education should integrate both medical and social aspects when providing information. It should extend beyond the classroom and better include men and boys, families, community members, and policy-makers [66]. Recent studies have found that boys’ knowledge of menstruation is often quite limited, but they also point out that boys were sympathetic, curious to learn more, and eager to support menstruating girls [43]. Education alone will not solve all problems related to menstrual health conditions; however, it is an important step to raise awareness, normalize menstruation, and enable menstruators to seek care when necessary.

Menstrual literacy should be recognized as part of sexual and reproductive health education [67]. Discussing menstruation is an essential part of understanding pubescent changes [68]. In some contexts, menarche is perceived as a sign of sexual or marriage readiness [69], and a lack of menstrual education could expose menstruators to sexual risk and leave them uninformed to make decisions about their bodies [68]. As Chris Bobel explains, “for girls to develop true agency at the site of their bodies, they must feel empowered to ask questions, evaluate options, and act in their own best interest, even if contrary to cultural norms. Doing this requires an authentic and agentic relationship with body, not one centered on discipline and control” [3]. The focus of menstrual education must shift from propagating bodily control and management rooted in gendered oppression to enabling bodily autonomy.

### Recognizing menstrual stigma as reflecting patriarchal power

Strengthening the agency and autonomy of menstruators requires recognizing the power inherent in menstrual stigma and understanding how such power is wielded to control menstruators’ bodies. Too often, an individualistic view perceives stigma purely as a social problem, that can be addressed through education, awareness-raising and changing attitudes alone [26]. Instead, we need to recognize that menstrual stigma is structurally embedded and intertwined with gendered oppression.

The efforts to engage cisgender men and boys reflect an acknowledgment that patriarchal power relations play a significant role in all contexts, and hegemonic masculinities privilege cis men as those with power [70]. Depending on the context, power relationships also fall along the lines of race, caste, gender identity, and other identifiers. Many menstruating individuals face compounded stigmas that enable and reinforce each other. Trans and nonbinary menstruators, in particular, are often marginalized and excluded [71, 72]. Rather than addressing their needs, menstrual policies often perpetuate a problematic gender binary that conflates men/boys as non-menstruators and women/girls as default menstruators, effectively leaving out menstruators who are not cis women. One of the policies that most extensively addresses the role of men and boys is the Kenyan MHM Policy which “seeks to ensure that myths, taboos and stigma around menstruation are addressed by providing women, girls, men and boys with information on menstruation.” The policy views such information as necessary for everyone in society in order to “ensure that *women and girls menstruate* in a safe and hygienic environment” [14, emphasis added], thus labelling women and girls as the default and, in fact, only menstruators. Similarly, the Indian National MHM Guidelines reference the important role of men and boys, but only with the goal of supporting girls and women: “In order to ensure that adolescent girls and women have the necessary support and facilities, it is important that the wider society, communities and families must challenge the status quo and break the silence around menstruation” [42]. None of the policy documents we reviewed address the menstrual needs of trans and non-binary individuals (while the Indian Guidelines for Swachh Bharat Mission refer to “the third-gender” and that they “should be allowed to use the [sanitation] facility of their choice (men or women)” [45]. As a result, the policy documents perpetuate a cis-normative, binary construction of gender and ignore the needs of trans and non-binary menstruators.

With regard to engaging society at large, many existing policies vaguely seek to break the silence and present menstrual products as a “stigma-management solution.” As a result, they ignore the power inherent in menstrual stigma. Stigma is more than silence. Stigma is multilayered and affects menstruators’ lives in different ways—from the way they are treated by others to the way they view themselves. The perception of menstruators and menstruation is influenced by “harmful gender norms, gender inequality, and related power structures in households and institutions, as well as by a lack of awareness, knowledge, and understanding about menstruation” [1]. Merely talking about menstruation does not address these power structures; and breaking the silence does not dismantle menstrual stigma.

At its core, stigma stems from unequal power relations. Bruce Link and Jo Phelan use the term “stigma power” to “refer to instances in which stigma processes achieve the aims of stigmatizers with respect to the exploitation, management, control or exclusion of others” [73]. Imogen Tyler conceptualizes stigma as a “material force, a structural and structuring form of power … that is written on the body and gets under the skin” [26]. It serves to discipline and exercise social control; it is deliberately employed to hide menstruators away and keep them down through the means of shame and low self-esteem. As Alexandra Brewis and Amber Wutich argue, “By defining those with less power as unsanitary, hygiene stigmas can be all-too-easily deployed for the political and economic gain by those who already have it” [36]. The dangers of stigma power are exacerbated through internalization: “People who are disadvantaged by the exercise of symbolic power are often influenced, sometimes without realizing it, to accept cultural assessments of their value and rightful (lower) place in the social order” [73]. Menstruators thus risk being stigmatized not only externally but also internally, viewing themselves as “less” due to their menstrual status.

The power of menstrual stigma is exercised invisibly by defaulting to the needs of the non-menstruating body and not accommodating the needs of the menstruating one. Awareness raising may be the first step to address previously overlooked needs. Yet, even when those in power receive menstrual education, attitudes and perceptions of menstruation as “dirty” often persist, signaling the need for more in-depth engagement on menstrual stigma and its effects [74, 75]. Menstrual stigma and its inherently oppressive power run deep; in many instances, menstrual needs are not overlooked but deliberately ignored. In the United States, for example, 27 states still charge value-added tax on menstrual products despite widespread campaigns on the inherent injustice of the tax [76, 77]. This demonstrates a lack of political will, not a lack of awareness. Stigma power must be addressed head-on to dismantle it.

To undo menstrual stigma, we must address these gendered power relations. Deepa Rao et al. note that “stigma is inherently a cross-sectoral phenomenon and thus efforts to reduce stigma and its pernicious effects require a multi-level approach” [78]. This includes addressing not only individual levels but also “incorporat[ing] community-, organizational-, and structural-level influences” [78]. We need to disentangle the workings of menstrual stigma and its impacts in multiple spheres of life, including health, education, work, and public life. This requires developing a better understanding of the lived experiences of menstruators and how they experience stigma; it requires changes in medical school curricula and trainings to teach healthcare providers not to dismiss menstrual pain; it requires understanding how the perception of menstruators as menstruators influences economic opportunities and participation in public life. Identifying the workings of menstrual stigma and the power it holds—such as in the case of a call center operator in the United States who was fired because her heavy bleeding “soiled” the carpet—is the first step [79]. Menstrual stigma permeates every facet of society. Rather than managing menstruation (away), efforts to raise awareness and break the silence provide an opportunity to expose menstrual injustices across all spheres of life, which then provides the basis for more comprehensive policy development. We might be able to break the silence around menstruation, but stigma rooted in misogyny and gendered oppression cannot be broken as easily. It needs to be actively challenged, dismantled, and undone.

### Limitations

Our study faced a number of limitations. First, it only focused on policy efforts by governments, while various nonstate actors have driven many developments in the menstrual health and hygiene space through their advocacy and services [3]. In addition, we only examined four countries. We made this decision based on a global overview of policy developments and selected countries that were early adopters of policies and represented a range of geographic contexts. Notably, we did not include any countries from Latin America because most policy developments in the region are more recent than in Africa, South Asia and North America.

Travel restrictions due to COVID-19 meant that all participants needed reliable internet connections, since introductions were facilitated via email and interviews were conducted remotely. Moreover, the different governance structures across countries led to a slight variation in the number of individuals belonging to each stakeholder group. In Senegal, half of our interviewees were governmental officials, whereas in the other countries about a quarter of interviewees were governmental, with the rest belonging to civil society. Nonetheless, we were able to collect extensive data in all four countries that provide deep insights into policy-making.

## Conclusion

Across the four countries, policy-makers and advocates identified stigma as a key challenge for meeting menstrual needs. Policies reflected this by seeking to tackle stigma through awareness raising and education. They sought to break the silence around menstruation, and many initiatives did so quite loudly—in small convenings and large policy forums, in various forms of media, and through engaging an ever-larger number of organizations and individuals. We do not mean to dismiss these efforts—they are essential for raising awareness. However, going back to the question we asked at the beginning—whether menstrual policies succeed in disrupting the status quo of the pernicious control of menstruators’ bodies—the answer is “no.” Policies recognize stigma, but they do not contribute to dismantling it.

Certainly, we cannot expect policies alone to undo stigma; but policies can and should play a crucial role in normalizing and destigmatizing menstruation (rather than further entrenching it, which some do inadvertently). The priorities they define, the language they choose, and the framing they adopt matter. What emerges to date is a story of missed opportunities and an apparent fear among policy-makers and advocates to consider what tackling menstrual stigma would actually mean. The funding for informational and educational initiatives under the Swachh Bharat Mission and the SABLA guidelines promoting comprehensive menstrual literacy in India, the menstrual health education law in New York, and the “MHM plus” initiative in Kenya were all steps in the right direction, but they were not used to their full potential; and even funding allocated toward education remained underutilized. Tangible outcomes such as products and facilities drowned out educational initiatives.

Most strikingly, policy-makers and advocates seemed unprepared to accept that breaking the silence means that “there will be blood” [80]. They did not seem ready to make menstruation visible. Menstruators and society at large receive mixed messages: “Yes, let’s talk about menstruation. But please do so in a ‘respectable’ way.” Policies address menstruation (or rather “MHM”) in public, but menstruators are told to manage in private. Bleeding itself is to be hidden, and menstrual hygiene initiatives continue to focus on management and concealment. The omnipresent pad served to keep menstruation “under wraps” [28]. Current policies accommodate menstrual stigma rather than menstrual needs. By reverting to menstrual products, hygiene, and management, policies and initiatives assume that menstrual stigma can (and must) be hidden, but that means it will persist in its invisible space—the space where it is most powerful.

Policy-makers and advocates seem scared of (or, more likely, unwilling to face) what might change if they actually did what they set out to do. It is easier to fall back into the same patterns that do not challenge the power of stigma. On the positive side, initiatives sought to engage extensively with men and boys, implicitly recognizing patriarchal power relations in households, schools, and governments. However, tackling the invisible power of stigma and its impacts in all spheres of life requires working with healthcare providers, educators, employers, public officials, and others in position of power.

Shifting the focus of menstrual education from bodily control to bodily autonomy would be a significant step in empowering menstruators to make decisions about their bodies and to advance gender justice. Comprehensive menstrual health education—education that enables menstruators to determine their “normal,” that covers biological and sociocultural dimensions, and that seeks to ensure informed decision-making—is the best antidote to stigma. Stigma can only persist in the context of misinformation and darkness, which is why efforts to raise awareness are essential and need to go further.

The recommendations that emerge from this analysis are as clear as they are complex: Make use of existing opportunities—they are usually hard-won and should be used to push for further change. Recognize that the power of stigma is strongest where it is invisible. Ensure that policies do not inadvertently reinforce stigma by focusing on hygiene and concealment. Use language that normalizes menstruation. Promote comprehensive menstrual literacy and recognize that information is power. Dismantling menstrual stigma requires shifting gendered power dynamics and moving from a perception of menstruators as “lesser than” to ensuring that their menstrual status does not prevent them from exercising power in their personal, social, and public lives.

## Supporting information

S1 Checklist Questionnaire on inclusivity in global research. (DOCX)

S1 Table Policy documents in India, Kenya, Senegal, and the United States. (DOCX)

S1 Text Interview guide in English. (PDF)

S2 Text Interview guide in French. (PDF)
