# Supplementary material for: The persistent power of stigma: A critical review of policy initiatives to break the menstrual silence and advance menstrual literacy
Source: PLOS Glob Public Health. 2022 Jul 14;2(7):e0000070. doi: 10.1371/journal.pgph.0000070 (PMC10021325; doi:10.1371/journal.pgph.0000070)
Supplement: S1 Table — (DOCX) [file pgph.0000070.s002.docx]

**Supporting Information**

**S1 Table. Policy documents in India, Kenya, Senegal, and the United States (n=34).**

| **Country** | **Year** | **Document Title** | **Institution** | **Overall Objective** | **Specific Focus on Menstruation** |
| --- | --- | --- | --- | --- | --- |
| India | 2009 | National Mission for Secondary Education  (Rashtriya Madhyamik Shiksha Abhiya) | Ministry of Human Resource Development | Increase the quality of and access to secondary education | Recognizes need for MHM infrastructure in secondary schools |
| India | 2011 | Rajiv Gandhi Scheme for Empowerment of Adolescent Girls / SABLA Guidelines | Ministry of Women and Child Development | Ensure the nutritional, hygienic, reproductive, and sexual health of adolescent girls | Recognizes how menstruation interacts with nutrition and health, addresses common health problems, beliefs, and stigma |
| India | 2013 | Modification in Total Sanitation Campaign  (Nirmal Bharat Abhiyan) | Ministry of Drinking and Water Sanitation | Improve sanitation in rural regions, end open defecation | Allows funds to go towards raising awareness, MHM education, and the development of MHM infrastructure in schools and other public facilities |
| India | 2014-2019 | Clean India Mission (Rural)  (Swachh Bharat Abhiyan - Gramin) | Ministry of Drinking Water and Sanitation and Ministry of Urban Development | Achieve a clean and open-defecation-free India | Seeks to guarantee MHM infrastructure in schools, environmental disposal, and awareness-raising/education |
| India | 2015 | Menstrual Hygiene Management National Guidelines | Ministry of Drinking and Water Sanitation | Resource for state and local level stakeholders to support girls and women and ensure adequate MHM | Seeks to provide adolescent girls with MHM choices; improve MHM infrastructure in schools, including safe disposal; and disseminate materials to different stakeholders |
| India | 2016 | National Adolescent Health Programme  (Rashtriya Kishor Swasthya Karyakram, or RKSK) | Ministry of Health and Family Welfare | Ensure holistic development of adolescents, particularly marginalized populations | Seeks to increase access to quality products, safe and environmentally conscious disposal, and awareness-raising via a Menstrual Hygiene Scheme (MHS) |
| India | 2017 | Guidelines on Gender Issues in Sanitation | Ministry of Drinking and Water Sanitation (part of Swachh Bharat Abhiyan) | Recognize the roles of women and third-gender people in issues of sanitation | Provides a list of key points to consider in the construction of MHM-sensitive facilities, including safe disposal, and emphasizes the need to dismantle menstrual stigma |
| India | 2018 | Operational Guidelines on School Health Programme  (Ayushman Bharat Pradhan Mantri Jan Arogya Yojana) | Ministry of Health and Family Welfare | Promotes and monitors student health | Calls for the provision of menstrual pads in schools, collects data on girls’ counselling on menstrual hygiene knowledge |
| India | 2018 | Removal of VAT on Sanitary Pads | Goods and Services Tax Council | Repeal the value-added tax on sanitary pads | n/a |
| India | 2019 | Mass Medicine Distribution Scheme for India  (Pradhan Mantri Bhartiya Janaushadhi Pariyojana) | Department of Pharmaceuticals | Ensures affordability of quality medicines | Offers biodegradable menstrual pads at reduced rates |
| Kenya | 2004 | Removal of VAT on Sanitary Pads | Parliament of Kenya | Repeal the value-added tax on sanitary pads | n/a |
| Kenya | 2011 | Zero-rated import duty for sanitary towels | Parliament of Kenya | End import duty on sanitary pads | n/a |
| Kenya | 2011 | Sanitary Towels Programme | Department for Gender in the Ministry of Public Service and Gender | Provide menstrual pads for all school-going girls in public primary and secondary schools | n/a |
| Kenya | 2016-2030 | Kenya Environmental Sanitation and Hygiene Policy | Ministry of Health | Improve access to and quality of sanitation services | Acknowledges menstruation as a key indicator of health; ensures a safe environment with sufficient MHM infrastructure; affirms the need for product provision and the education of stakeholders |
| Kenya | 2017 | Basic Education (Amendment) Act | Parliament of Kenya | Address school absenteeism due to menstruation via the provision of menstrual pads and safe disposal in schools | n/a |
| Kenya | 2018 | School Health Policy | Ministry of Education, Ministry of Health | Address the psychosocial and health needs of learners in and out of schools | Reaffirms product provision and MHM infrastructure in schools; addresses how menstruation intersects with concerns of human rights, nutrition, and school absenteeism; establishes key indicators to collect on menstrual hygiene |
| Kenya | 2018-2022 | Ministry of Public Service, Youth and Gender Affairs State Department for Gender Affairs – Strategic Plan | State Department for Gender Affairs | Promote gender equality | Reaffirms goal of product provision for school-going girls |
| Kenya | 2019-2030 | Menstrual Hygiene Management Policy & Strategy | Ministry of Health | Improve MHM for girls and women in Kenya | Seeks to break the silence, address stigma, increase access to products, ensure safe disposal, and recognizes MHM as a human rights issue |
| Kenya | 2020 | Kenya Standard – KS 2925: 2020 Reusable sanitary towels specification | Kenya Bureau of Standards | Create regulatory guidelines for manufacturers of reusable menstrual pads | n/a |
| Senegal | 2014-2018 | Strategic Plan for the Sexual and Reproductive Health of Youth  (Plan stratégique de santé sexuelle et de la reproduction des adolescente(e)s/jeunes) | Ministry of Health and Social Action  (Ministère de la Santé et de l’Action sociale) | Improve sexual and reproductive health for adolescents | Acknowledges menstruation as a risk factor for anaemia |
| Senegal | 2016-2025 | Sectoral Policy Letter for Development  (Lettre de Politique Sectorielle de Développement) | Ministry of Water and Sanitation  (Ministère de l’Eau et de l’Assainissement) | Advance the management of water and sanitation services | Recognizes the lack of MHM-specific infrastructure; discusses the successes and failures of integrating MHM into the sector and increasing awareness among WASH practitioners |
| Senegal | 2016 | National Strategy for Gender Equity and Equality  (Stratégie National pour l’Équité et l’Égalité de genre) | Ministry of Health and Social Action  (Ministère de la Santé et de l’Action sociale) | Dismantle discrimination based on gender | Recognizes the need for special accommodations for menstruation in women’s prisons |
| Senegal | 2016 | Plan for the Institutionalization of Gender  (Plan d’institutionnalisation du genre) | Ministry of Water and Sanitation  (Ministère de l’Eau et de l’Assainissement) | Institutionalize considerations of gender and ensure equitable access to ministry services | Seeks to prepare MHM training modules for ministry staff |
| United States | 2005 | Menstrual Tampons and Pads: Information for Premarket Notification Submissions (510(k)s)—Guidance for Industry and FDA Staff | Food and Drug Administration | Recommendations for preparing premarket notification submissions for the safety of menstrual pads and tampons | n/a |
| United States | 2016 | Emergency Food and Shelter Program Responsibilities and Requirements Manual | Federal Emergency Management Agency | Support people in long-term economic emergencies | Includes menstrual products as eligible items to be covered with funding |
| United States | 2017 | Provision of Feminine Hygiene Products | Department of Justice, Federal Bureau of Prisons | Provide menstrual products to all female federal prisoners | n/a |
| United States | 2018 | First Step Act | United States Congress | Improve criminal justice outcomes and reduce the federal prison population | Requires free, ample, and quality product provision for all federal prisoners |
| United States | 2020 | **Coronavirus Aid, Relief, and Economic Security (CARES) Act** | United States Congress | Provide economic relief to individuals, families, and businesses in response to the COVID-19 pandemic | Allows certain accounts’ funds to be used for menstrual products on an employer and individual level |
| United States: New York State | 2016 | Exempting Feminine Hygiene Products from Sales Taxes | New York State Assembly | Exempt menstrual products from state sales tax | n/a |
| United States: New York State | 2019 | Menstrual Products Right to Know Act | New York State Assembly | Require menstrual product manufacturers to disclose the ingredients of their products on labels | n/a |
| United States: New York State | 2019 | Bill on Informational Materials Concerning Menstrual Disorders | New York State Assembly | Prepare informational materials for dissemination to schools, patients, healthcare practitioners | Contains information on importance of menstrual health, signs and symptoms of menstrual disorders, treatment options for endometriosis and other disorders |
| United States: New York City | 2016 | New York City Council Intro-1122A, Law Number 2016/082: Menstrual Products in Correctional Settings | New York City Council | Issue menstrual products for inmates | n/a |
| United States: New York City | 2016 | New York City Council Intro 1123-A, Law Number 2016/083: Menstrual Products in Shelters | New York City Council | Provide a sufficient supply of menstrual products in various city shelters | n/a |
| United States: New York City | 2016 | New York City Council Intro 1128-2016, Law Number 2016/084: Provision of Feminine Hygiene Products in Schools | New York City Council | Provide menstrual products at no cost to students in school bathrooms | n/a |
