# Supplementary material for: The persistent power of stigma: A critical review of policy initiatives to break the menstrual silence and advance menstrual literacy
Source: PLOS Glob Public Health. 2022 Jul 14;2(7):e0000070. doi: 10.1371/journal.pgph.0000070 (PMC10021325; doi:10.1371/journal.pgph.0000070)
Supplement: S1 Text — (PDF) [file pgph.0000070.s003.pdf]

Brief Background

More and more countries are developing legislative and policy frameworks on menstrual hygiene and health. Policies cover different aspects such as de-taxing menstrual products, information and education campaigns, and ensuring provision of menstrual products to specific population groups.

Amidst building momentum, advocates for menstrual health and hygiene are looking at these policy developments as markers of progress with significant expectations. Yet, there are also risks of adopting policies that are narrow in scope and as a result may not de-stigmatize menstruation to the extent possible. It is therefore an opportune time to reflect on recent policy developments.

We seek to explore the processes, triggers, drivers and opportunities that have led to the elevation of menstrual health at the policy level. We are particularly interested in whose voices, interests and needs are centered and whose are marginalized in these policies and the processes leading to their adoption, and how this influences the framing of policies both in terms of their scope and the targeted populations.

We will therefore conduct a process-oriented review informed by the human rights principles of participation, non-discrimination and substantive equality, and accountability as well as substantive human rights guarantees in three selected countries: India, Kenya, and the United States. In doing so, we will conduct qualitative in-depth in person interviews, focus groups and desk research. Based on this review, we are interested in compiling lessons learnt and identifying the levers that drive menstrual hygiene and health policies grounded in human rights considerations.

Columbia University IRB
IRB-AAAS8659 (Y01M00)
IRB Exemption Date: 02/18/2020

Semi-structured Interview Guide for Menstrual Health Policy Review

My name is Purvaja S Kavattur and I am working with the Institute for the Study of Human Rights at Columbia University on a menstrual health policy review project. This project will run over the next year. With policies on menstrual hygiene and health being adopted in countries across the world, we seek to explore the processes, triggers, drivers and opportunities that have led to the elevation of menstrual health at the policy level. We are particularly interested in whose voices, interests and needs are centered and whose are marginalized in these policy processes. This interview will run between 50-80 minutes.<br><br>Consent process

The semi-structured interview will be informed by the below questions. The questions will be chosen from below list, based on context and subject matter expertise. Depending on responses there may be further follow-up questions.

Introduction Questions:

1. Clearly state your name, organization and role.
2. What does menstrual hygiene and health mean to you?
   Probes:
   - What is needed for good menstrual hygiene and health?
   - Individual practices v. societal practices?
3. What is your connection to menstrual hygiene and health policy?
   Probes:
   - What prompted you to first get involved in this way?
   - When was this?
   - Who brought you into this work?

Trigger for Action Stage Questions:

1. Menstrual hygiene and health is an emerging policy field. How has the policy field changed over the past five years?
   Probes
   - What are the key events that occurred in the past five years?
   - local, state, national and international level key events?
   - What are the key policies that emerged from this period?
2. What factors catalyzed this policy action?
   Probes
   - For each policy event: Which actors were instrumental? Social Movements, NGOs, civil society organizations, government organizations, media outlets, etc?
   - Of these factors – which is the single most important?
   - Would you characterize these factors as top-down, bottom-up, community oriented, grassroots?

Columbia University IRB
IRB-AAAS8659 (Y01M00)
IRB Exemption Date: 02/18/2020

Policy Development Stage Questions: (repeat this stage for every key policy they mentioned)

1. You mentioned that in the past five years "X,Y,Z" policies emerged. I'm interested in the formal development of these policies. What were the key elements taken into consideration by governments during the policy design?
   Probes
   - Which aspects of menstruation was addressed?
   - What health disparities / gaps did these policy address?
   - Timeline? Funding? Ministries?
   - Were there alternatives considered?
2. What specific aspects of menstruation were addressed?
   Probes
   - How was this chosen as a priority area? Consider the people involved.
   - Were other aspects discussed?
3. What are the policies stated objectives?
4. What steps did policy-makers take to inform themselves of menstrual hygiene and health needs and preferences on the ground?
   Probes
   - Was there formative research conducted? Situational / contextual analysis?
   - Consultations? National and international?
5. Who was consulted and/or involved in the policy development process?
   Probes
   - Which ministries were involved?
   - Consultations within government?
   - What other non-governmental stakeholders?
   - Who was not consulted?
6. For this policy what were the target populations? Why?
   Probes
   - Which populations do you think this policy reached the easiest?
   - Which populations were not included? Why?
   - Are there populations that you believe are particularly marginalized / excluded?
   - Are there specific ministries that can best address this particular population?
7. Are there populations that you believe are particularly marginalized / excluded?
   Probes
   - What specific barriers does this population face?
   - Does this policy particularly address the needs of X population?

Policy Implementation Strategies Questions:

1. An important aspect of the policy process is implementation. I am interested in understanding the different implementation frameworks governments use when devising policies. Based on XYZ policies what implementation strategies are put in place?
   Probe
   - What are the specific means of implementation? Sensitization trainings, curricula, budgetary allocations, institutional involvement?
   - Do these strategies differ by ministry?
   - What challenges have you faced with implementation?

Columbia University IRB
IRB-AAAS8659 (Y01M00)
IRB Exemption Date: 02/18/2020

2. What monitoring frameworks has the government adopted?

   Probe

   - What are the criteria and indicators?
   - What resources are allocated towards monitoring and evaluation?
   - How do these frameworks differ by ministry?

3. Menstrual hygiene and health is increasingly recognized as having intersectoral needs. In what ways are different ministries held accountable?

   Probe

   - Is menstrual health and hygiene integrated into different ministries' budgets?
   - What about non-financial accountability mechanisms?

Closing Questions:

1. What would be one small thing you could change about menstrual hygiene and health policy landscape be? Why?
2. Now, thinking without any constraints (financial, political, bureaucratic). What is one thing you would like to radically change about the menstrual hygiene and health policy landscape? Why?
3. Are there specific documents, organizations, or people we should reach out to?

   Probes

   - Why?

4. Is there anything else you would like to add?

Thank you for your time!

Columbia University IRB
IRB-AAAS8659 (Y01M00)
IRB Exemption Date: 02/18/2020
