# Supplementary material for: The persistent power of stigma: A critical review of policy initiatives to break the menstrual silence and advance menstrual literacy
Source: PLOS Glob Public Health. 2022 Jul 14;2(7):e0000070. doi: 10.1371/journal.pgph.0000070 (PMC10021325; doi:10.1371/journal.pgph.0000070)
Supplement: S2 Text — (PDF) [file pgph.0000070.s004.pdf]

Guide d’entretien

**Bref historique**

De plus en plus de pays élaborent des politiques sur l'hygiène et la santé menstruelles. Les politiques couvrent différents aspects tels que la détaxe des produits menstruels, l'information et l'éducation et la fourniture de produits menstruels à des groupes de population spécifiques.

Les défenseurs de la santé et de l'hygiène menstruelles examinent ces politiques et développements comme des marqueurs de progrès en termes d’égalité des sexes, avec des attentes importantes. Pourtant, il existe également des risques d'adopter des politiques ayant une portée limitée et, par conséquent, ne réussiraient pas à exploiter toutes les possibilités permettant éliminer la stigmatisation portée sur les menstruations. C'est donc le moment opportun pour réfléchir aux récents développements politiques.

Nous cherchons à explorer les processus, déclencheurs, moteurs et opportunités qui ont conduit à l'élévation de la santé menstruelle au niveau politique. Nous sommes particulièrement intéressés par les voix, les intérêts et les besoins qui sont formulés et ceux qui sont marginalisés dans ces politiques ainsi que les processus menant à leur adoption. Nous sommes également intéressés par la manière dont ces éléments influent sur l’élaboration des politiques, sur leur portée et sur les populations ciblées.

Nous procéderons donc à un examen axé sur les processus, fondé sur les principes des droits humains, de la participation, la non-discrimination et l'égalité réelle, la responsabilité ainsi que sur les garanties des droits humains dans les quatre pays sélectionnés: l'Inde, le Kenya, le Sénégal et les États-Unis. Ce faisant, nous mènerons des entretiens qualitatifs approfondis avec des personnes individuelles, des groupes de discussion et des recherches documentaires. Sur cette base, nous sommes intéressés à compiler les leçons apprises et à identifier les leviers qui conduisent à l’élaboration de politiques sur la menstruation, l'hygiène et la santé fondées sur des considérations relatives aux droits humains.

**Guide d'entretien semi-structuré pour l'examen des politiques de santé menstruelle**

Je m'appelle Purvaja S Kavattur et je travaille à l'Institut pour l'Étude des Droits Humains à l’Université Columbia sur un projet d'examen des politiques de santé menstruelle. Ce projet se déroulera au cours de l’année 2020. Avec l’augmentation croissante du nombre de politiques publiques sur l'hygiène et la santé menstruelle adoptées dans les pays du monde entier, nous cherchons à explorer les processus, déclencheurs, moteurs et opportunités qui ont conduit à l'élévation de la santé menstruelle au niveau politique. Nous sommes particulièrement intéressés par les voix, les intérêts et les besoins qui sont formulés et ceux qui sont marginalisés dans ces politiques ainsi que les processus menant à leur adoption. Nous sommes également intéressés par la manière dont ces éléments influent sur l’élaboration des politiques, sur leur portée et sur les populations ciblées. Cet entretien durera entre 50 et 80 minutes.

Columbia University IRB
IRB-AAAS8659 (Y01M02)
IRB Exemption Date: 04/29/2020

**Processus de consentement**

L'entretien semi-structuré sera informé par les questions ci-dessous. Les questions sont choisies dans la liste ci-dessous, en fonction du contexte et de l'expertise en la matière. L'enquêtrice peut poser d'autres questions de suivi en fonction des réponses obtenues.

**Questions d'introduction:**

1. Indiquez clairement votre nom, votre organisation et votre rôle.
2. Que signifie pour vous l'hygiène et la santé menstruelle?

Les questions de sondage

- Selon vous, de quoi a-t-on besoin pour une bonne hygiène et santé menstruelles?
- Pratiques individuelles contre pratiques sociétales?

3. Quel est votre lien avec la politique d'hygiène et de santé menstruelle?

Les questions de sondage

- Qu'est-ce qui vous a poussé à vous impliquer de cette façon?
- À quel moment avez-vous décidé de vous impliquer dans ce processus ? Jusqu'à quand ? Etes-vous toujours impliqué dans la mise en œuvre de la politique ?
- Qu'est-ce qui vous a motivé à vous impliquer dans ce travail ?

**Questions pour l'étape déclencheur:**

1. L'hygiène et la santé menstruelle est un domaine politique émergent. Comment le paysage politique a-t-il changé les cinq dernières années?

Les questions de sondage

- Quels sont les événements clés qui se sont produits au cours des cinq dernières années?
- Événements clés au niveau local, régionale, national et international?
- Quelles sont les politiques clés qui ont émergé de cette période?

2. Quels facteurs ont catalysé cette action politique?

Les questions de sondage

- Pour chaque événement politique: Quels acteurs ont joué un rôle? Mouvements sociaux, ONG, organisations de la société civile, organisations gouvernementales, médias, etc.?
- De ces facteurs - quel est le plus important?
- Considéreriez-vous ces facteurs comme top down (émanent des décideurs vers les communautés/individus), bottom up (émanant des communautés/individus vers les décideurs) ou mené par les communautés et/ou individus ?

**Questions sur l'étape de l'élaboration des politiques: (répétez cette étape pour chaque politique clé mentionnée)**

Columbia University IRB
IRB-AAAS8659 (Y01M02)
IRB Exemption Date: 04/29/2020

1. Vous avez mentionné qu'au cours des cinq dernières années, des politiques «X, Y, Z» ont émergé. Je suis intéressé par le processus formel de développement de ces politiques. Quels ont été les éléments clés pris en considération par le gouvernement lors de la conception de la politique?

Les questions de sondage

- Quels aspects de la menstruation sont abordés?
- Quelles disparités / lacunes ces politiques santé/hygiène menstruelle ont-elles comblées?
- La chronologie ? Le financement ? Les ministères ?
- Des alternatives ont-elles été envisagées? Si oui, lesquelles ?

2. Quels aspects spécifiques de la menstruation ont été abordés?

Les questions de sondage

- Comment cela a-t-il été choisi comme domaine prioritaire? Considérez les personnes impliquées.
- D'autres aspects ont-ils été discutés?

3. Quels sont les objectifs déclarés des politiques?

4. Quelles mesures les décideurs ont-ils/elles prises pour s'informer des besoins en matière d'hygiène et de santé menstruelle et préférences de la population?

Les questions de sondage

- Des recherches formatives ont-elles été menées? Analyse situationnelle / contextuelle?
- Consultations? National et international?

5. Qui a été consulté et / ou impliqué dans le processus d'élaboration des politiques?

Les questions de sondage

- Quels ministères étaient impliqués?
- Consultations au sein du gouvernement?
- Quels autres acteurs non gouvernementaux?
- Qui n'a pas été consulté?

6. Pour cette politique, quelles étaient les populations cibles? Pourquoi?

Les questions de sondage

- Quelles populations pensez-vous que cette politique a atteint le plus facilement?
- Quelles populations n'ont pas été incluses? Pourquoi?
- Y a-t-il des populations qui, selon vous, sont particulièrement marginalisées / exclues?
- Y a-t-il des ministères spécifiques qui peuvent mieux répondre à cette population particulière?

7. Y a-t-il des populations qui, selon vous, sont particulièrement marginalisées / exclues?

Columbia University IRB
IRB-AAAS8659 (Y01M02)
IRB Exemption Date: 04/29/2020

Les questions de sondage

- À quels obstacles spécifiques cette population est-elle confrontée?
- Cette politique répond-elle particulièrement aux besoins de la population X?

**Questions sur les stratégies de mise en œuvre des politiques:**

1. Un aspect important du processus politique est la mise en œuvre. Je suis intéressé à comprendre les différents cadres de mise en œuvre que les gouvernements utilisent lors de l'élaboration des politiques. Basé sur politiques XYZ quelles stratégies de mise en œuvre sont mises en place?

Les questions de sondage

- Quels sont les moyens spécifiques de mise en œuvre? Formations de sensibilisation, curricula, allocations budgétaires, implication institutionnelle?
- Ces stratégies diffèrent-elles selon le ministère?
- Quels défis avez-vous rencontrés lors de la mise en œuvre?

2. Quels cadres de suivi le gouvernement a-t-il adoptés?

Les questions de sondage

- Quels sont les critères et indicateurs?
- Quelles ressources sont allouées au suivi et à l'évaluation?
- En quoi ces cadres diffèrent-ils selon le ministère?

3. L'hygiène et la santé menstruelles sont de plus en plus reconnues comme ayant des besoins intersectoriels. Comment les ministères rendent-ils compte de leurs actions et/ou résultats ?

Les questions de sondage

- La santé et l'hygiène menstruelles sont-elles intégrées dans les budgets des différents ministères?
- Qu'en est-il des mécanismes de responsabilité non financière?

**Questions de clôture:**
Quelle sont les changements que vous souhaitez voir au sujet de la politique d'hygiène et de santé menstruelle ?

1. Quelle serait la petite chose que vous souhaiteriez voir changée au sujet de la politique d'hygiène et de santé menstruelle? Pourquoi?

2. Maintenant, penser sans aucune contrainte (financière, politique, bureaucratique). Quelle est une chose que vous souhaitez changer radicalement dans les politiques d'hygiène et de santé menstruelle? Pourquoi?

3. Existe-t-il des documents, des organisations ou des personnes spécifiques que nous devrions contacter?

Columbia University IRB
IRB-AAAS8659 (Y01M02)
IRB Exemption Date: 04/29/2020

Les questions de sondage

- Pourquoi?

4. Y a-t-il autre chose que vous aimeriez ajouter?

*Merci pour votre temps!*

Columbia University IRB
IRB-AAAS8659 (Y01M02)
IRB Exemption Date: 04/29/2020
